# Uncommon Factors Leading to Nephrotic Syndrome

**DOI:** 10.3390/biomedicines13081907

**Published:** 2025-08-05

**Authors:** Ljiljana Bogdanović, Ivana Babić, Mirjana Prvanović, Dragana Mijač, Ana Mladenović-Marković, Dušan Popović, Jelena Bogdanović

**Affiliations:** 1 Institute of Pathology, School of Medicine, University of Belgrade, 11000 Belgrade, Serbia; 2 School of Medicine, University of Belgrade, 11000 Belgrade, Serbia; 3 Emergency Center, University Clinical Center of Serbia, 11000 Belgrade, Serbia; 4 Clinic for Gastroenterology and Hepatology, University Clinical Center of Serbia, 11000 Belgrade, Serbia; 5 Department for Gastroenterology and Hepatology, University Hospital Center Dr Dragiša Mišović-Dedinje, 11000 Belgrade, Serbia; 6 Clinic for Endocrinology, Diabetes and Metabolic Diseases, University Clinical Center of Serbia, 11000 Belgrade, Serbia

**Keywords:** nephrotic syndrome, rare causes, syndromes

## Abstract

Nephrotic syndrome (NS) is characterized by proteinuria, hypoalbuminemia, edema, and hyperlipidemia. Apart from the traditional causes of NS, such as minimal change disease, focal segmental glomerulosclerosis, diabetes, infections, malignancies, autoimmune conditions, and nephrotoxic agents, there are also rare causes of NS, whose knowledge is of the utmost importance. The aim of this article was to highlight the less well-known causes that have a significant impact on diagnosis and treatment. Genetic syndromes such as Schimke immuno-osseous dysplasia, familial lecithin-cholesterol acyltransferase deficiency with two clinical variants (fish-eye Disease and the p.Leu364Pro mutation), lead to NS through mechanisms involving podocyte and lipid metabolism dysfunction. Congenital disorders of glycosylation and Nail–Patella Syndrome emphasize the role of deranged protein processing and transcriptional regulation in glomerular injury. The link of NS with type 1 diabetes, though rare, suggests an etiology on the basis of common HLA loci and immune dysregulation. Histopathological analysis, particularly electron microscopy, shows mainly podocyte damage, mesangial sclerosis, and alteration of the basement membrane, which aids in differentiating rare forms. Prompt recognition of these novel etiologies by genetic analysis, renal biopsy, and an interdisciplinary panel is essential to avoid delays in diagnosis and tailored treatment.

## 1. Introduction

The presence of an increased amount of protein in the urine, called proteinuria, hypoalbuminemia, and edema, are the main features of nephrotic syndrome (NS). Minimal change disease (MCD) and focal segmental glomerulosclerosis (FSGS), as primary, and diabetic nephropathy (DN) and immune diseases, as secondary, are just some of the well-known and described causes of NS [1,2,3,4]. However, in addition to these causes, there are also some rare causes of NS, the understanding of which is extremely important not only for establishing an accurate diagnosis, but also for the application of more effective therapy.

When we talk about the genetic reasons for the occurrence of NS, we believe that it is important to highlight the role of genetic predisposition in its development. Numerous studies have indicated hereditary diseases that cause the development of NS by causing damage to the glomerular basement membrane (GBM) or by affecting various proteins that are extremely important for the function of different parts of the nephron [5,6]. Alport syndrome (AS) is certainly one of the examples of these diseases. AS is a genetic disorder characterized by the presence of mutations in genes responsible for the synthesis of type IV collagen, which is an integral part of basement membranes. If we keep this in mind, it is clear that GBM damage will occur in the kidney, which will further cause hereditary nephritis, which is clinically manifested by proteinuria (a sign of NS), microhematuria (the presence of blood in the urine), and later progression to chronic renal insufficiency (HBI) [5,6,7,8].

In addition to these hereditary diseases, it is certainly important to mention other genetic diseases in which changes in the genes associated with the function of podocytes—specialized cells, whose damage is responsible for the passage of larger molecules and urine, and therefore for the occurrence of proteinuria at the nephrotic level—have been shown. Namely, we know well that podocytes play a key role in the creation and maintenance of the glomerular filtration barrier (GFB). The GBM serves as a barrier for proteins in vivo. In order to be able to perform its function, the GBM also needs filtration cracks that are responsible for preventing the passage of albumin from the capillary lumen into the urinary space [9]. The glycocalyx, which is made of polysaccharides bound to proteins located on the outer surface of the cell membrane, builds glycoproteins and glycolipids, passes the filtration clefts, covers the endothelial cells and restricts the passage of macromolecules, and therefore ensures that plasma albumin does not participate in GFB for the most part [2]. This actually means that the filtration clefts, together with GBM and fenestrae, play a very important role in the selective permeability of GFB, but it is assumed that the filtration cleft is the weakest link because mutations of the filtration cleft proteins are significantly associated with the occurrence of proteinuria [10,11,12]. Previous research has shown that mutations in the proteins of the filtration clefts, including podocin and nephrin, lead to the loss or disruption of the filtration clefts and the retraction of the foot process extensions of podocytes [13]. Therefore, subsequent research indicated genetic variations, namely mutations in the NPHS1 and NPHS2 genes, which encode proteins necessary for good podocyte function, which are significantly associated with the development of NS [14,15,16,17].

Long ago it was shown that, although rare, infections can cause NS. Infection of the upper respiratory tract caused by Streptococcus is often discussed as the cause of NS. Specifically, primarily in children, kidney damage can occur after a history of streptococcal infection, manifested by NS, which is a disease designated as post-infectious NS. The cause of damage to kidney structures and, consequently, the cause of NS, is other infections, among which we are mainly talking about infections with the hepatitis virus. In particular, damage to the kidney structures has been described in the case of infection with the hepatitis B virus, which is characterized by changes in the glomeruli, known as membranous nephropathy (MN). In addition to the aforementioned infections, human immunodeficiency virus (HIV), cytomegalovirus, and parvovirus B1 infections are also cited as secondary causes of NS [18,19,20].

Malignant diseases, such as lymphoma and leukemia, can cause NS through different mechanisms. Immune responses caused by neoplastic tissues can lead to kidney damage. Synthesis of monoclonal light chains contributes to the development of NS in hematological malignancies, including multiple myeloma, which can potentially result in the development of tubular nephropathy or renal amyloidosis. Amyloidosis, which is characterized by the deposition of amyloid protein in various tissues and organs, can cause NS by the accumulation of these proteins within the interstitium of the kidney and glomeruli [21,22].

Systemic diseases, such as systemic lupus erythematosus (SLE), an autoimmune disease where the body’s immune system mistakenly attacks its own tissues, can also lead to NS in some cases. In these conditions, the underlying disease process may cause kidney damage, leading to the characteristic proteinuria and other symptoms of NS [23,24].

Metabolic disorders, primarily diabetes mellitus (DM), are important factors that can cause NS. Over time, DN can also manifest itself as NS, especially with inadequate glycemic control. Recent studies have shown that glycemic variability and duration of diabetes are correlated with the severity of kidney disease [25,26,27,28].

Another unusual but documented factor in the development of NS is the use of certain drugs. Medications, such as non-steroidal anti-inflammatory drugs (NSAIDs), lithium, and certain chemotherapeutic agents, have been associated with the development of NS. The exact mechanisms by which these drugs may cause NS have not been fully elucidated. It is believed that the occurrence of NS during their use may occur due to direct toxicity to the glomeruli or changes in the immune system [29,30,31].

Common causes of NS are well documented, but it is also important to consider rare causes that may ultimately change treatment strategies and patient outcomes. Therefore, the aim of this paper is to investigate and describe the rare causes of NS. A detailed review of the literature will analyze factors, as well as specific disorders, that can cause damage to renal structures and manifestations of characteristic symptoms of NS, such as proteinuria, edema, and hyperlipidemia.

## 2. Definition, Epidemiology, and Diagnosis of Nephrotic Syndrome

NS is a clinical condition whose most important manifestations are excessive loss of protein in the urine (proteinuria), swelling, and low levels of albumin in the blood, i.e., hypoalbuminemia [1].

Based on the underlying pathophysiological mechanisms, NS can be divided into three groups: (1) congenital/infantile nephrotic syndrome (CNS), (2) steroid-sensitive nephrotic syndrome (SSNS), and (3) steroid-resistant nephrotic syndrome (SRNS). The CNS is characterized by the appearance of proteinuria in the first three months of life. In contrast, NS that becomes apparent later, i.e., in the first year of life, more precisely between four and twelve months, is identified as infantile NS, while NS that manifests after that is called childhood NS, which, based on the response to corticosteroid treatment observed four weeks after therapy, can be further divided into SSNS and SRNS [32]. With technological advances in next-generation sequencing, the approach to NS has changed. Previously, genetic forms of NS were considered rare disorders [33]. However, contemporary research has shown that at least 66% of cases with SRNS occur during the first year of life [17] and that up to 30% of cases of SRNS with onset before the age of 25 are attributed to an underlying monogenic defect [8]. In familial NS and childhood-onset NS, this proportion escalates to between 57% and 100%, in contrast to 10% to 20% for sporadic childhood-onset cases. Despite advances in genetics, significant uncertainties remain regarding the pathogenesis of SRNS of unidentified genetic origin and SSNS [16]. The effectiveness of glucocorticoids and other immunosuppressive agents in certain cases of SRNS and in all cases of SSNS indicates a significant involvement of the immune system in the pathogenesis of these conditions. However, recent research suggests that immunosuppressive therapies may have a direct effect on podocytes, in addition to their immunological effects.

The annual incidence of NS is from 1.15 to 2.1 new cases per 100,000 children [2,34]. Although it is noted to occur more often in boys than in girls, this difference disappears in older age groups. NS occurs more frequently in Japan and among children of Asian descent compared to the European pediatric population. Studies suggest that genetic factors play a significant role in the occurrence of NS. In addition, some studies have indicated that its occurrence may vary among different ethnic groups [35,36]. Some of the articles published in the scientific literature reveal that both genetic and environmental factors can influence the appearance of this clinical condition. This is supported by articles that showed that in Nigeria, as many as 80% of children diagnosed with NS have a form that responds very well to corticosteroid therapy, which is very similar in Asian and European pediatric populations. In support of the view that both genetic and environmental factors influence the disease, there are also studies conducted in France that recorded a seasonal jump in the occurrence of this clinical condition, which was attributed to infections as possible triggers. This is similar to the observation of a study conducted in the United States, which found a relationship between the occurrence of NS and allergies and asthma [34,37].

When it comes to the diagnosis of NS, it is important to mention that it differs significantly in adults and children. Specifically, when signs of NS are noted in the adult population, the diagnostic process usually begins with a kidney biopsy with subsequent histopathological classification. On the other hand, when it comes to the pediatric population in the diagnostic procedure, doctors first monitor the response to the applied corticosteroid therapy for several weeks. After a certain period of time, NS is classified based on the clinically recorded response to corticosteroids. Thus, it is stated that in about 80% of children with NS, eight weeks after starting corticosteroid therapy, they went into remission. Histopathological findings indicated that, in approximately 93% of cases of children with NS that respond well to corticosteroid therapy, the cause of NS is MCD, while in about 7% there is another glomerular pathology. Light microscopic analysis of kidney tissue samples with a histopathological finding of MCD does not show any visible histopathological changes, so the diagnosis of MCD is made based on the electron microscopic analysis of these samples. Electron microscopy reveals significant destruction of podocyte foot extensions, which indicates podocyte injury. Unfortunately, in addition to cases that show a good response to the use of corticosteroid therapy, there are also cases, about 20% of children, which do not respond to the use of corticosteroid therapy. In children with NS resistant to corticosteroids, histopathological analysis of kidney tissue samples shows FSGS, a condition that indicates permanent damage to podocytes [38]. MCD and FSGS are often referred to as related conditions, but some cases labeled as MCD may eventually develop into FSGS. It is not clear whether these are simply different stages of the same problem or completely separate diseases [2,39].

Due to the limitations of standard diagnostic methods, rapid identification of NS usually presents challenges. More precise and faster diagnoses are made possible by advanced molecular and diagnostic methods. Because quantification of protein excretion can be obtained either from a 24 h urine sample or by analyzing the protein/creatinine ratio from the first morning urine sample, urinalysis serves as the basic first step. Blood tests are used to determine albumin levels, lipids, and indicators of kidney function. Microscopic analysis of kidney tissue obtained by biopsy can define the nature and extent of damage. In cases where the disease has a genetic basis, such as congenital or steroid-resistant forms of nephrotic syndrome, genetic testing can find certain mutations. Further understanding of the immune components of the disease comes from the discovery of autoantibodies, such as anti-PLA2R, which may be helpful in monitoring therapeutic responses. One possible indicator of the disease is the expression of the CD80 molecule on podocytes. High expression of CD80 on podocytes could be associated with NS and could be used as a diagnostic tool. Especially in kidney cells, such as glomeruli and podocytes, the study of gene expression patterns (transcriptome profiling) can help reveal certain molecular mechanisms associated with NS. The integration of these molecular and diagnostic tools helps in the rapid and correct diagnosis of the disease, which enables swift and adequate medical treatment [40,41,42,43,44].

The diagnostic approach requires, in addition to a careful history and physical examination, laboratory tests. One of them is the urinalysis, which includes the qualitative or quantitative measurement of microalbuminuria, that is, the detection of proteinuria and examination for microscopic hematuria and effusion. Blood tests evaluate serum albumin, cholesterol, creatinine and other relevant parameters. It is necessary to perform the following blood tests to distinguish nephrotic syndrome caused by vasculitis or connective tissue disease from other causes: antinuclear antibodies, double-stranded DNA antigens, selective antibodies to extractable nuclear antigens, complement factors (C3, C4), antinuclear cytoplasmic antibodies, as well as high-sensitivity C-reactive protein. To determine the underlying cause of NS, a kidney biopsy is necessary in adult patients, while it is less commonly performed in the pediatric population [45].

## 3. Clinical Presentation

A characteristic sign of NS is massive proteinuria, usually defined as urinary protein excretion of more than 3.5 g per day in adults or 40 milligrams per square meter of body surface area per hour in children [45]. The glomerulus, a filtration body located within the renal corpuscle, consists of a layer of endothelial cells interspersed with fenestrated cells and a basement membrane, while Bowmans capsule consists of podocytes and squamous cells. Between the podocytes there is a slit for filtering, which is the most important part in preventing the passage of proteins. Specifically, this layer is responsible for the selective leakage of proteins. In addition to the previously mentioned causes of damage to podocytes that are responsible for the occurrence of proteinuria, one of the main signs of NS, it has been proven that the increase in hydrostatic pressure within the glomerulus causes increased permeability, which increases the amount of protein passing through the mentioned barrier [1].

Hypoalbuminemia, i.e., a reduced level of serum albumin, is also noted in patients with NS, which is caused by albuminuria, which is caused by increased glomerular permeability. Hypoalbuminemia causes a decrease in oncotic pressure, which further causes an increase in transcapillary water filtration in the body. When the amount of filtered fluid exceeds the maximum lymphatic flow, which will occur due to low intravascular oncotic and high capillary hydrostatic pressure, edema develops. Edema can manifest itself in different ways. Specifically, edema can manifest as mild periorbital swelling or it can manifest as generalized. It should be emphasized that the appearance of edema is often the first sign of NS. The severity of edema is almost always correlated with the degree of proteinuria and hypoalbuminemia [46,47,48,49]. Otherwise, the described changes, in NS, will lead to a decrease in plasma volume and, secondarily, to an increased retention of sodium and water by the kidneys [48,49].

NS causes hyperlipidemia and changes in lipid metabolism, as a result of which the levels of serum cholesterol, triglycerides and lipoproteins are increased, while the concentration of HDL remains unchanged or is only slightly increased, and the ratio of total cholesterol to HDL cholesterol is increased. Research has shown that within NS, the severity of hyperlipidemia follows the severity of proteinuria. It is believed that lipid abnormalities are caused by changes in the expression and activity of proteins that play an important role in the synthesis, transport, remodeling, and catabolism of lipids and lipoproteins. Elevated levels of lipids and lipoproteins in NS significantly affect the development and progression of kidney and cardiovascular diseases [45,50,51].

NS can progress to chronic renal failure or End-Stage Renal Disease (ESRD), where the underlying cause certainly plays a significant role. The progression of NS to ESRD is particularly prominent in SRNS or when it is based on FSGS. Several factors increase the risk of progression from NS to ESRD, namely, in addition to response to therapy, persistent proteinuria, the presence of hypertension, and coexisting conditions such as diabetes and obesity. Literature data indicate that proteinuria is the main risk factor for the progression of kidney disease and suggest that patients with NS who have high levels of proteinuria have a higher risk of accelerated decline in renal function. In addition, poorly controlled blood pressure and diabetes can exacerbate kidney damage, suggesting the need for comprehensive treatment approaches [52,53].

Proteinuria directly causes damage to the renal tubules as they reabsorb filtered protein, which can further lead to inflammation, cellular dedifferentiation, and fibrosis in the interstitium. Nephron loss gradually occurs, as a result of which numerous compensatory mechanisms are triggered, including hyperfiltration, which is, an increase in the rate of filtration. This, although beneficial initially, ultimately contributes to further damage. All this can lead to an increase in pressure in the glomeruli themselves, which will damage the sensitive glomerular capillaries. Tubules also adapt to increased filtration by increasing their load, which further leads to hypoxia in them. Then, the damaged tubular epithelial cells can transform into myofibroblasts, which lead to fibrosis and significant destruction of the renal structure. It is important to mention that inflammation, which is caused by proteinuria and/or other factors, releases mediators that further worsen fibrosis. It is completely clear that in the end there is a progressive decline in kidney function, i.e., that they lose the ability to filter waste products and regulate fluid and electrolyte balance, which ultimately results in the development of chronic renal failure or ESRD [52,53,54,55].

Other clinical manifestations vary depending on the underlying cause and severity of the disease.

## 4. Rare Causes of Nephrotic Syndrome

### 4.1. Schimke Syndrome: Pathophysiology and Histopathological Changes Linked to Nephrotic Syndrome

Schimke’s immune-bone dysplasia (SIOD) is a rare, autosomal recessive disease characterized by numerous clinical manifestations, such as impaired growth, spondyloepiphyseal dysplasia, a defect in T-cell immunity, and SRNS. Studies have shown that among patients with genetically determined NS, SIOD occurs in 2.4–9.4% of cases [56,57,58].

#### 4.1.1. Pathophysiology

SIOD is a disorder caused by mutations in the SMARCAL1 gene, which is known to be involved in chromatin remodeling and gene expression crucial for kidney development and function. In order to understand the role of SMARCAL1 in the development of renal damage in patients with SIOD, studies initially followed, by immunohistochemical methods, the expression of SMARCAL1 in both developing and mature kidneys. These studies showed SMARCAL1 expression in the metanephric mesenchyme and urothelial epithelium, but also in all stages of nephron formation during development in the fetal kidney. Postnatally, SMACAL1 expression was also observed in tubules, podocytes and endothelial cells. In addition, using TUNEL analysis of kidney biopsies from patients with SIOD, research has shown that there is an increase in DNA fragmentation. It was also pointed out that the enzymatic activity of SMARCAL1 is very important in DNA replication and repair, as well as in the process of DNA-nucleosome restructuring and chromatin remodeling during the regulation of gene expression. The assumption that DNA damage already starts during fetal development is based on the observed expression pattern of SMARCAL1 during fetal kidney development. It is this DNA damage that is believed to cause the observed DNA fragmentation and disruption of genomic integrity. All this indicates that SMARCAL1 loss of function leads to genomic instability associated with DNA replication that causes impaired renal cell function, primarily podocytes. The role of the SMARCAL1 gene during kidney development suggests that its dysfunction may disrupt the normal formation of nephrons and glomeruli, contributing to FSGS, a change observed in patients with SIOD during histopathological analysis of kidney biopsies [59,60,61,62]. Recent research indicates the existence of podocytic infolding glomerulopathy (PIG) in SIOD patients. Although the mechanisms of the formation of PIG have not yet been clarified, it has been observed that kidney function and proteinuria improve with the use of corticosteroid therapy. This knowledge led the researchers to assume that, in these cases, immune abnormalities are responsible for the occurrence of proteinuria. However, it is not supported since in many patients neither autoimmune disorders nor abnormal complement levels were found. This study further points out that PIG was found in patients with a mutation in the SMARCAL1 gene, suggesting that mutations in the SMARCAL1 gene impair the structure and function of glomeruli, especially podocytes [60].

#### 4.1.2. Histopathological Changes

In patients with SIOD, signs of FSGS are typically found by histopathological analysis of renal tissue biopsy. In the pathogenesis of FSGS, damage or loss of podocytes is most significant. The previously mentioned studies that analyzed the expression of SMARCAL1 in the kidney, using special staining and electron microscopy, categorized FSGS into different morphological variants: (1) the apex variant, which is characterized by the finding of adhesions in the regional apex, although they can be found in places other than the perihilar region, and (2) the collapsing variant of FSGS, which is characterized by the finding of only one glomerulus with segmental or global collapse and podocyte hyperplasia, which does not respond well to therapy and can rapidly lead to end-stage renal disease (TBI). Studies have shown that the variant type of FSGS can progress to a collapsing form. They also observed that different SMARCAL1 gene mutations are associated with different types of FSGS, and that lesion progression or mutational variability affect clinical outcomes, thus furthering the understanding of FSGS in SIOD [59,63,64,65].

#### 4.1.3. Clinical Implications and Therapeutic Interventions

The clinical presentation includes, in addition to symptoms of kidney involvement, characteristic growth disorders (low growth) and a weakened immune system. Skeletal abnormalities (spondyloepiphyseal dysplasia) affecting the spinal vertebrae and hips are characteristic; as a result, these patients have a short trunk, a short neck, and a protruding stoma (due to lumbar lordosis). Hyperpigmentation of the macula and neurological problems (migraine, stroke) can also occur. Renal involvement is manifested by NS that is usually resistant to corticosteroid therapy, progressing to ESRD [66,67,68]. Based on a review of the medical literature, it can be seen that nephropathy usually develops before the age of 12 and progresses to ESRD within the next 1 to 11 years [69].

The diagnosis of Schimke syndrome with NS requires a multifaceted approach that includes clinical evaluation, radiological imaging and genetic testing. Characteristic features of spondyloepiphyseal dysplasia will be revealed by examining the skeleton and X-ray imaging, while immunological tests will enable the diagnosis of immune dysfunction. To diagnose NS, a urine analysis is necessary, and confirmation of kidney involvement is obtained after the analysis of kidney biopsy samples. However, the gold standard for confirming the diagnosis of this syndrome is certainly the identification of mutations in the SMARCAL1 gene through next-generation sequencing [70,71].

Treatment requires a comprehensive and multidisciplinary approach and is aimed at managing symptoms, minimizing complications, and preserving renal function. Immunosuppressive agents, such as corticosteroids, cyclosporine, and tacrolimus, are commonly used to reduce proteinuria and preserve renal function. However, the effectiveness of these agents may be limited in some patients, and long-term use may lead to significant side effects, including infections and nephrotoxicity. In cases of severe or refractory NS, more aggressive interventions, such as rituximab or cyclophosphamide, may be considered. In addition, in these patients, suppurative therapy is necessary. As a potential therapy for Schimke syndrome associated with severe immune dysfunction and progressive kidney failure, hematopoietic stem cell transplantation is also reported [72,73,74].

#### 4.1.4. Future Directions and Research

As we can conclude from the aforementioned reviews of the medical literature so far, it is necessary to increase awareness of Schimke syndrome and improve early diagnosis. Certainly, understanding the genetic and molecular basis of Schimke syndrome holds promise for the development of targeted therapies. The research community must focus on further investigating the molecular mechanisms linking SMARCAL1 gene deficiency to podocyte dysfunction in order to advance pharmacotherapy. We believe that targeting pathways involved in podocyte integrity could be the basis of future therapies aimed at preserving renal function [56,74,75].

### 4.2. Lecithin-Cholesterol Acyltransferase (LCAT) Deficiency

A key role in cholesterol esterification and HDL metabolism is played by the enzyme lecithin cholesterol acyltransferase, which is bound to high (HDL)- and low (LDL)-density lipoprotein particles. LCAT deficiency is caused by mutations in the LCAT gene located on chromosome 16 (16q22). Deficiency of this enzyme is a rare hereditary syndrome that is caused by impaired HDL metabolism, which further leads to abnormal lipid profiles. Several studies have investigated the mechanisms by which NS arises in cases of LCAT deficiency. Initially, these studies investigated the effect that NS has on cholesterol metabolism in the liver. Later, they began to study the esterification and absorption of cholesterol from peripheral tissues. Numerous studies followed, focusing on the molecular basis of triglyceride-rich lipoprotein abnormalities caused by NS. What all studies have in common is the finding of decreased expression of hepatic triglyceride lipase and lipoprotein lipase in animals that had NS. It was shown that animals with NS had a significantly reduced concentration and enzyme activity of LCAT in plasma, in addition to its normal expression in the liver observed in animals with nephrotic syndrome, and it was suggested that this reduction was accompanied by a large loss of this enzyme through the urine. Thus, the findings of these studies suggest that urinary losses of this enzyme contribute to LCAT deficiency, which plays a significant role in the pathogenesis of impaired HDL-mediated reserve cholesterol transport in NS [50,51,76,77].

There are two clinical variants of LCAT deficiency: (A) fish-eye disease (FED)—the disease occurs due to the partial absence of LCAT enzyme activity, and (B) the familial form of LCAT deficiency—the disease occurs due to the complete absence of LCAT enzyme activity [51,77].

#### 4.2.1. Fish-Eye Disease: Pathophysiology and Histopathological Changes Linked to Nephrotic Syndrome

Fish-eye disease—FED, a rare disorder characterized by the accumulation of unesterified cholesterol in various tissues, such as the cornea, kidneys, and other organs: it is believed that the accumulation of non-esterified cholesterol can lead to a number of clinical manifestations, including the development of NS [78,79,80,81].

##### Pathophysiology

From the conducted research, it can be assumed that the accumulation of non-esterified cholesterol can interfere with the normal function of GFB, which allows the passage of proteins of large molecules, such as albumin, into the urine. In addition, findings suggest that cholesterol deposits can lead to structural and functional changes in the glomeruli that contribute to the development of NS [78,80,82,83,84].

##### Histopathological Changes

Research that analyzed kidney biopsies found that people suffering from FED have changes in the kidneys that are associated with the accumulation of non-esterified cholesterol, which include the presence of foam cells within the glomeruli and in the interstitium. These foam cells originate from macrophages that are loaded with lipids, i.e., non-esterified cholesterol. In glomeruli, GBM thickening, proliferation of mesangium, both mesangial cells and matrix can be observed by light microscopy, while lipid vacuoles in endothelial cells can be seen by electron microscopy. These histopathological changes can probably lead to a disturbance of the normal filtration function of the glomeruli which can contribute to the development of proteinuria. Furthermore, several studies, based on immunofluorescence analysis of kidney samples, have revealed deposition of glycosylceramide within the glomerulus. This finding provides further support for the role of lipid accumulation in the pathogenesis of NS in FED. It is very important to point out that there is variability in the severity of renal involvement—some patients with FED show only mild proteinuria, while others develop severe, progressive renal disease leading to end-stage renal failure [80,82,83,85,86,87].

##### Clinical Implications and Therapeutic Interventions

The clinical presentation is different, and one of the manifestations is NS, the onset and severity of which can vary, whereby in some patients NS progresses to ESRD. Patients with FED are characterized by corneal opacity, which is caused by the deposition of lipids in the corneal stroma, which can significantly damage vision and, in severe cases, require a corneal transplant. A low level of HDL cholesterol is also characteristic, and dysfunctional HDL particles that are less efficient are a potential accelerating factor of the atherosclerotic process. Some of the patients may have splenomegaly, hepatomegaly, or lymphadenopathy [81,88,89].

The diagnosis of FED must involve a combination of clinical assessment, biochemical testing, and histopathological analysis of tissue samples. Suspicion of the existence of FED is certainly caused by the findings of corneal clouding, low HDL-cholesterol, and proteinuria. Measurement of LCAT activity, HDL cholesterol level and other lipid parameters, urinalysis can confirm the diagnosis, while histopathological analysis of kidney biopsy reveals characteristic glomerular damage, as well as other histological changes. Genetic testing, which identifies a mutation in the LCAT gene, is extremely important for confirming the diagnosis of FED [80,81].

Treatment options for FED have been quite limited, but advances in gene therapy (directed to the liver to introduce a functional copy of the LCAT gene) and therapeutic enzyme replacement (potential replacement of the missing LCAT enzyme) represent promising avenues. Dietary interventions, traditional therapies (use of angiotensin-converting enzyme inhibitors (ACE) or angiotensin receptor blockers (ARB)), which reduce proteinuria and inflammation and protect the kidney, statins are necessary. In severe cases of NS, immunomodulatory therapy is also considered, which could simultaneously improve changes in the eyes. In patients with end-stage renal failure, kidney transplantation is also considered, but it is believed that it would not be very successful considering that the underlying metabolic disorder persists after transplantation [80,81,83,84].

##### Future Directions and Research

Future research should focus on developing targeted therapies that address the underlying LCAT deficiency in FED. Enzyme replacement therapy, in which recombinant LCAT is administered to patients, as well as gene therapy, which aims to correct the LCAT gene mutation, are just some of the promising approaches. In addition, new technologies in gene editing, especially CRISPR-Cas9, may offer opportunities to correct mutations associated with fish-eye disease. Conducting clinical trials of such innovations could provide valuable insights into the future of personalized medicine and its application in this rare disorder. It is necessary to further investigate the mechanisms by which lipid deposition leads to glomerular damage and fibrosis. Moreover, longitudinal studies are needed to evaluate the long-term outcomes of patients with FED [80,83,84].

#### 4.2.2. p.Leu364Pro Mutation Familial Lecithin Cholesterol Acyl Transferase Deficiency: Pathophysiology and Histopathological Changes Linked to Nephrotic Syndrome

Familial deficiency of lecithin cholesterol acyltransferase (LCAT) is a rare, autosomal recessive inherited disorder characterized by the complete absence of LCAT enzyme activity in plasma. The p.Leu364Pro mutation found is a specific genetic variant associated with a complete lack of LCAT enzyme activity, which often manifests with significant clinical consequences, including NS [78,90,91,92,93,94].

##### Pathophysiology

Research has shown that the lack of the LCAT enzyme is the cause of impaired esterification of cholesterol within HDL particles, which leads to several consequences. Non-esterified cholesterol that accumulates in HDL causes a reduction in the size of HDL particles and thus leads to changes in lipoproteins which, as such, are deposited in various organs, including the kidneys. The exact mechanisms by which lipid accumulation impairs GFB integrity are not fully understood, but are believed to involve inflammation and oxidative stress, which contribute to podocyte damage and increase glomerular permeability, resulting in proteinuria. Regarding the already-mentioned p.Leu364Pro mutation, it is emphasized that it particularly affects the structure and function of the enzyme; how it affects its catalytic activity and probably contributes to the severity of the phenotype. It is also reported that the p.Leu364Pro mutation may be associated with a more severe form of LCAT deficiency and a higher likelihood of developing NS compared to other mutations. The specific impact of this mutation on protein structure and function may contribute to a more pronounced disorder of lipoprotein metabolism and subsequent kidney damage [75,76,93,94,95,96,97].

##### Histopathological Changes

Light microscopic analysis of kidney biopsies shows thickening of the capillary walls and a bubbly appearance of the basal membrane, expanded mesangium, and variable infiltrate of the mesangium, with foam cells that can still be seen in the blood vessels and in the interstitium. Electron microscopic analysis of the samples revealed lacunae in the basement membranes and electron-dense deposits in the mesangium, with completely obliterated foot-like extensions of podocytes [89,97,98].

##### Clinical Implications and Therapeutic Interventions

The clinical presentation of patients with p.Leu364Pro mutation and LCAT deficiency include the presence of a series of symptoms, such as corneal opacity caused by lipid deposition in the cornea, hemolytic anemia resulting from abnormal lipid status, and proteinuria, specifically NS. The onset and severity of NS can vary, but often leads to progressive kidney damage and end-stage renal disease if left untreated. Suspicion of LCAT deficiency should certainly be raised by the presence of these clinical signs, together with family history [78,99].

The diagnosis of familial deficiency of LCAT, especially with the p.Leu364Pro mutation, involves a combination of clinical evaluation, biochemical evaluations, and genetic testing. Biochemical analyses reveal reduced or absent activity of the LCAT enzyme in plasma; low levels of HDL and elevated levels of cholesterol and triglycerides. A definitive diagnosis is confirmed by genetic testing that identifies specific mutations such as the p.Leu364Pro mutation, in the LCAT gene. Histopathological analysis of kidney biopsies can assess the extent of glomerular damage and confirm the presence of lipid deposits. As LCAT deficiency is inherited in an autosomal recessive manner, the integration of family history and clinical picture certainly helps in the diagnosis. In the early detection of this condition, and therefore in its treatment, the screening of the population at risk and the family can play a significant role [78,100,101,102].

There is currently no specific *treatment* for this condition. Therefore, treatment focuses on alleviating symptoms and complications. It is necessary to modify the diet, which includes reducing fat intake and using statins and other lipid-lowering agents, although they have not been shown to be completely effective in treating dyslipidemia associated with LCAT deficiency. For patients with signs of NS, corticosteroids are the first line of treatment, followed by ACE inhibitors or ARB. In severe forms of NS with progressive kidney damage, dialysis and kidney transplantation can be considered. Research into the development of novel therapies, such as plasmapheresis and gene therapy, has gained momentum, and progress in these areas promises future treatment paradigms [99,103,104].

##### Future Directions and Research

Future research should focus on understanding the mechanisms involved in renal lipid deposition and the development of NS in LCAT deficiency due to the p.Leu364Pro mutation. This may facilitate the development of targeted therapies to prevent or reverse kidney damage. Enzyme replacement therapy, where recombinant LCAT enzyme is administered to patients, holds promise but faces challenges related to enzyme delivery and stability. Gene therapy, aimed at correcting the LCAT gene mutation, is another potential approach but is still in the early stages of development. Understanding the precise mechanisms by which the p.Leu364Pro mutation affects LCAT enzyme function and lipid metabolism may lead to the identification of new therapeutic targets [76,105,106,107].

### 4.3. Nephrotic Syndrome Co-Existing with Type 1 Diabetes: Pathophysiology and Histopathological Changes

Coexistence of early NS with type 1 diabetes mellitus (T1D) is extremely rare [25,108,109,110].

#### 4.3.1. Pathophysiology

It is well known that in long-term T1D, hyperglycemia initiates a whole cascade of biochemical reactions leading to DN, a condition that can be induced by NS. Hyperglycemia causes oxidative stress and generation of advanced glycation end products, which contribute to nephron damage. In diabetes, the progressive loss of podocytes is responsible for the increased permeability, which allows larger molecules, such as albumin, to pass into the urine. This loss of selectivity is related to the development of NS. In addition, it has been shown that upregulation of inflammatory cytokines in people with diabetes significantly worsens glomerular damage, which further leads to proteinuria. Glomerulosclerosis is another very important feature of DN [28,111,112,113].

Despite this knowledge, there are limited data explaining the co-occurrence of NS and diabetes. Cases of children who developed NS very soon after the diagnosis of T1D have been described. On the other hand, cases of children who developed T1D after the diagnosis of idiopathic NS have also been recorded. As already emphasized, it is known that patients suffering from T1D develop proteinuria in advanced stages of the disease, usually 12 years after the diagnosis of T1D. Cases with a short duration of T1D and absence of organ (kidney) damage indicate the existence of non-diabetic nephropathy. Such cases are detected by finding an increased rate of glomerular filtration [26,27]. The simultaneous occurrence of NS and T1D is rare, but has been described for a long time, and some studies have highlighted the immunological basis for its occurrence. This attitude is explained by the finding of the simultaneous presence of NS and TD1 in children in whom either positivity for human leukocyte antigen (HLA) antigens A24, DR4 and DR53 or only the presence of DR44 was found. The presented findings suggest that steroid-sensitive NS and T1D may share the same HLA loci, which we know carry a genetic predisposition to these diseases. As a possible explanation for the simultaneous occurrence of NS and T1D, immune dysregulation is mentioned—where it is pointed out that the simultaneous occurrence of these conditions can be a reflection of a broader immune dysregulation that affects both the pancreas (in T1D) and the kidneys (in NS). These studies, however, raise the question of whether the appearance of NS in the context of T1D is the first manifestation of diabetic nephropathy or whether it could be another example of idiopathic NS, a condition with an unknown cause [26,27,28,113,114,115,116,117,118,119,120,121].

#### 4.3.2. Histopathological Changes

Studies that followed histopathological changes in kidney tissue samples obtained after biopsy from patients with concomitant NS and T1D mostly recorded the presence of MCD in the early stages of the disease. In advanced stages, light microscopic analysis of kidney tissue samples can reveal significant glomerular abnormalities, such as diffuse glomerulosclerosis, expansion of the mesangial matrix and thickened capillary walls. Electron microscopic analysis of these samples shows podocyte damage, which is another key feature in the histopathological findings. Deletion of foot-like extensions of podocytes is often observed in NS, and is especially pronounced in people suffering from diabetes. This deletion indicates a loss of podocyte function, further exacerbating GFB. The presence of glomerular hypertrophy and fibrosis may also be evident, visualized by specific staining techniques during histological examination [26,27,109,111,112,116,117,120,121].

#### 4.3.3. Clinical Implications and Therapeutic Interventions

The coexistence of NS and T1D has numerous *clinical challenges*. Patients with massive proteinuria are at increased risk of developing end-stage renal disease as well as cardiovascular disease due to the combination of macrovascular complications associated with diabetes and the hyperlipidemia and prothrombotic state associated with the nephrotic syndrome. In addition, it is necessary to mention that there is also a large psychological burden and that many patients experience anxiety and depression related to their condition, which can affect their adherence to treatment plans [23,26,28,109,116,122].

The diagnosis of NS in patients with T1D requires a comprehensive clinical evaluation that includes a detailed medical history, physical examination, and laboratory tests. Diagnosis begins with a urinalysis (detection of proteinuria), after which a 24 h urine collection can be performed to accurately quantify protein excretion. Laboratory analyses also include determining the level of serum albumin to assess the degree of hypoalbuminemia. Renal function tests are necessary to assess the degree of kidney involvement. Insight into kidney function and potential complications will be provided by GFR. In cases where the diagnosis is particularly difficult, a kidney biopsy may be warranted to determine the underlying pathology. Biopsy findings can help distinguish diabetic nephropathy from other causes of nephrotic syndrome, such as minimal change disease, which is relatively common in younger individuals [23,27,28].

The *treatment* of NS in the context of T1D requires a multifaceted approach, which includes glycemic control, where the cornerstone of treatment is insulin therapy, lifestyle changes, and pharmacological treatment. In addition to insulin therapy, agents such as glucagon-like peptide-1 (GLP-1) agonists and sodium-glucose co-transporter 2 (SGLT2) inhibitors, which also have effects on kidney protection, have been used recently to regulate glycemia. ACE inhibitors or ARBs are often used to treat hypertension and reduce proteinuria. In order to alleviate the symptoms associated with fluid overload and to reduce additional damage to the kidneys, dietary modification that includes reduced sodium intake and protein control is often advised [23,26,28,109].

#### 4.3.4. Future Directions and Research

Future research must certainly be aimed at uncovering and better understanding the common pathophysiological mechanisms underlying these two entities. It is necessary to find biomarkers for the early detection and progression of kidney disease. In addition, performing genetic studies could point to susceptible populations and advance personalized treatment strategies. Clinical research is also necessary to evaluate in detail the effectiveness of newer therapies in this dually diagnosed population. We believe that studies evaluating the long-term effects of glycemic control and renoprotective agents would provide significant data that would be of valuable help for further refinement of treatment strategies [27,109,116,123,124,125,126].

### 4.4. Congenital Disorders of Glycosylation: Pathophysiology and Histopathological Changes Linked to Nephrotic Syndrome

Rare multisystem hereditary metabolic diseases resulting from impaired synthesis, transport and processing of glycoproteins and glycolipids are classified in the group of congenital glycosylation disorders (CDG). It has long been well known that proteoglycans are present in the mesoderm, and that they are important for modulating tubule inductive signals with the help of integrins, which are glycoproteins. They make connections with components of the extracellular matrix that are important for cell adhesion. In basement membranes, glycoproteins are responsible for the adhesive support for the differentiation and migration of kidney cells. In addition, they can also participate in the formation of a physical barrier that is responsible for limiting cell–cell interaction. From such facts it can be concluded that glycosylation is necessary in maintaining the maturation of the nephron [126,127]. In addition, numerous previous studies have shown us that N-glycosylation involves the attachment of sugars to nucleotides, the stepwise assembly of oligosaccharide precursors, and the subsequent processing of glycans [127]. Therefore, glycosylation is a crucial process in which sugar molecules (glycans) are attached to proteins and lipids, which is necessary for the proper folding, transport, stability and function of proteins. It also plays a role in cell adhesion and signaling pathways [128,129,130,131]. If we keep all these functions and facts in mind, it is clear that defects in glycosylation can lead to a wide range of clinical manifestations, which can vary in severity and presentation depending on the specific affected gene, as well as the extent of the glycosylation defect [128,129,132,133]. In cases of CPG, the glycosylation process is disrupted, which can lead to misfolded or unfolded proteins. This can affect the proper assembly and function of proteins in various cellular processes, including those essential for adhesion and signaling [128,129,134]. Many cell surface proteins involved in cell–cell and cell–matrix interactions are glycosylated. Defective glycosylation in CDG can significantly impair these adhesion interactions, thereby contributing to developmental and neurological problems [128,129,135].

#### 4.4.1. Pathophysiology

As early as 1991, changes such as cystic and tubular proteinuria were described in patients with a syndrome then called carbohydrate-deficient glycoprotein syndrome, now the PMM2-CDG phenotype. Only after the description of these cases did the examination of possible kidney involvement in patients diagnosed with CDG begin, as changes that are part of multisystem involvement. Although research has shown that the CNS may be associated with CDG, only a few forms of CDG have explained some of the multiple mechanisms that contribute to the development of NS in these patients [126,128,136]. In particular, NS in infancy or early childhood has been observed in some subtypes of CDG, such as PMM2-CDG (phosphomannomutase 2-congenital disorder of glycosylation) and ALG1-CDG (ALG1-congenital disorder of glycosylation) [126,128,136,137]. One of the most common forms of CDG, which is caused by mutations in the phosphomannomutase 2 (PMM2) gene, has been observed to often involve the kidneys. The enzyme PMM2 is essential for the synthesis of mannose phosphate, which is an important component of N-linked glycosylation. Defective glycosylation, which results from mutations in the PMM2 gene, will affect the proper folding and function of numerous proteins, including those in the glomerulus. All this can lead to changes in the structure and function of the GBM, endangering the GFB. Other CDGs associated with renal involvement, i.e., the occurrence of NS, include ALG6 deficiency (designated as CDG-Ib) and ALG3 deficiency (designated as CDG-IIa), which affect the early steps in N-linked glycosylation, thereby affecting the structure and function of GBM proteins [126,128,136,137].

#### 4.4.2. Histopathological Changes

Histopathological examination of kidney biopsies from patients with CDG light microscopy reveals characteristic features consistent with NS. Kidney biopsies from cases of CDG with NS show diffuse mesangial sclerosis, which is a condition in which mesangial cells become thickened and sclerotic (hardened), which can lead to impaired glomerular filtration [136].

#### 4.4.3. Clinical Implications and Therapeutic Interventions

The spectrum of symptoms that occur in patients with this disorder is wide and includes edema, fatigue, abdominal pain, and increased susceptibility to infections. Developmental delay, liver dysfunction, and cardiac abnormalities occur in patients with CDG. Patients with CDG and NS have an increased risk of developing renal failure and mortality. Early recognition and treatment of NS are key to improving patient outcomes. Clinical manifestations of CDG, in addition to NS, are neurological disorders such as ataxia, stroke-like episodes, hepatomegaly, liver fibrosis, skeletal dysplasia, joint contractures and muscle weakness, heart problems (cardiomyopathy, pericardial effusion), eye abnormalities (strabismus, retinitis pigmentosa) [137,138,139,140].

The *diagnosis* of NS associated with CDG requires a multifaceted approach. Laboratory analyses are necessary to detect elevated serum protein levels and low serum albumin levels. That is, for findings that can indicate the existence of NS. A kidney biopsy is required to assess histopathological changes in the glomeruli and to rule out other causes of nephrotic syndrome. Genetic testing, such as whole exome sequencing or targeted gene sequencing, is necessary to identify the underlying genetic defect responsible for CDG. In addition, it is desirable to determine the pattern of glycosylation in serum and/or tissue samples using mass spectrometry, which increases the accuracy of the diagnosis [141,142,143].

Treatment focuses on preserving renal function and addressing the underlying metabolic defect. Symptomatic treatment may include dietary modification to reduce sodium and fluid intake, diuretics to relieve edema, intravenous albumin infusions to correct hypoalbuminemia, as well as immunosuppressive agents such as corticosteroids or calcineurin inhibitors. Enzyme replacement therapy and substrate inhibition strategies are being investigated, particularly for specific types of CDG. For example, in PMM2-CDG, attempts to provide alternative enzymatic pathways or correct metabolic disorders may be promising. In some types of CDG, targeted therapy is applied, for example, mannose supplementation (for CDG-Ib) and galactose supplementation [144,145,146].

#### 4.4.4. Future Directions and Research

Future research of CDG and NS should focus on several key areas. First, efforts should be focused on identifying new genes and pathways involved in glycosylation and their role in kidney function. There is also a great need for studies to elucidate the molecular mechanisms underlying glomerular damage in CDG-related NS. In addition, clinical trials are needed to evaluate the effectiveness of new therapeutic interventions, such as gene therapy or personalized medicine approaches. Long-term follow-up studies are also warranted to assess the natural course of CDG and NS and to identify predictors of disease progression [144,145,147,148].

### 4.5. Nail–Patella Syndrome: Pathophysiology and Histopathological Changes Linked to Nephrotic Syndrome

Nail–Patella Syndrome (NPS) is a rare hereditary autosomal dominant disorder that is primarily manifested by abnormalities in the development of nails, knees and elbows, i.e., nail dysplasia, hypoplasia or absence of the patella, deformity of the elbows. In patients with NPS, there are also clinical signs of eye and kidney involvement. Neurological diseases are also present [149,150,151].

#### 4.5.1. Pathophysiology

NPS is caused by a heterozygous mutation in the LMX1B gene, which is located on the long arm of chromosome 9 (9q34). This gene encodes an LIM-homeodomain transcription factor that is essential for the development of multiple organ systems, such as limbs, nails, and kidneys. Research has shown that mutations in the LMX1B gene disrupt the normal expression of genes involved in developmental processes, which further leads to the characteristic features of NPS. The precise mechanisms by which mutations in the LMX1B gene cause the various phenotypic manifestations of NPS are not fully elucidated, but disruption of multiple signaling pathways is believed to occur during development. The researchers believe that the pleiotropic effects during organogenesis attest to the broad regulatory function of LMX1B. Furthermore, research has pointed to the interaction between the homeodomain of the LMX1B gene and other genes as possible factors that can be significant in the development of many different conditions. Thus, they indicated the interaction of the LMX1B gene and the transcription factor PAX2, whose proteins are important for eye and kidney development [150,152,153,154,155,156].

The precise mechanism by which LMX1B mutations lead to kidney disease is not fully understood. So far we know that LMX1B has a very important role in podocyte development. Foot-like extensions of podocytes can be obliterated and show abnormal slit-like diaphragms, causing GBM splitting, as well as reduced endothelial fenestrations [157,158]. Bearing in mind these findings, as well as the already-mentioned interactions with other genes, it is assumed that altered expression of the LMX1B gene can affect the development and maturation of glomerular structures, making them susceptible to numerous damages. This may include altered expression of proteins crucial for glomerular filtration, extracellular matrix organization, as well as regulation of immune cells in the kidney [149,150,157,158].

#### 4.5.2. Histopathological Changes

Histopathological analysis of kidney biopsies, which involves examination of tissue samples by light, immunofluorescence and electron microscopy, from patients with NPS reveals characteristic histopathological changes. Light microscopic analysis of renal biopsies can show irregularly thickened GBM, which when viewed by electron microscopy appears moth-eaten due to deposition of collagen-like fibrillar material [158,159]. In addition, electron microscopy of kidney biopsy specimens showed ultrastructural findings associated with NPS-associated nephropathy, namely loss of podocyte foot-like extensions and slit-like diaphragm [158,159,160]. Specifically, the microscopic changes explain why patients with NPS can have proteinuria with or without hematuria [156,157].

#### 4.5.3. Clinical Implications and Therapeutic Interventions

The clinical presentation of NPS is characterized by deformities of the nails that are absent, hypoplastic or dysplastic (especially in the thumbs), and of the skeleton, where small or absent kneecaps can be seen, and elbow dysplasia. As part of NPS, the kidneys can also be affected, and the main renal manifestation is proteinuria, which can vary from mild asymptomatic to NS, and in some cases to terminal renal failure [149,154,159,161].

The *diagnosis* of NPS usually begins with a clinical examination, with characteristic findings on the nails and skeleton. X-rays of the knees, elbows, and pelvis are required to visualize these deformities. Genetic testing to identify mutations in the LMX1B gene, although not readily available, confirms the diagnosis. When nephrotic syndrome is suspected, a serum and urine analysis is required, and a kidney biopsy may be needed to assess kidney tissue changes [149,154,162,163].

There is no specific drug to treat the underlying genetic defect. *Treatment* of NPS with NS is aimed at alleviating symptoms and preventing complications. The first line usually involves the administration of corticosteroids, which effectively reduce proteinuria and can lead to remission in many cases. In addition, ACE inhibitors or ARBs are used to control proteinuria and blood pressure. In cases of end-stage renal failure, dialysis or a kidney transplant may be required. Other manifestations require pain relief with physiotherapy, wearing corsets, and, in some cases, surgery for joint or bone problems. Frequent controls are needed to monitor kidney function and assess the need for additional interventions [157,163,164,165].

#### 4.5.4. Future Directions and Research

The rarity of NPS presents challenges in research and treatment. It is very important to investigate the mechanisms by which LMX1B mutations lead to nephropathy. In addition, it is important to conduct studies to investigate the efficacy of newer immunosuppressive therapies or biological agents to optimize treatment approaches for NS associated with NPS. Genetic counseling and population studies could contribute to understanding the prevalence and long-term outcomes of NPS. It is necessary to research and improve gene therapy, as well as strategies targeting podocyte function, to protect the kidney. We believe that the priority was to work on the development of specific biomarkers for the early detection of NPS-related nephropathy, which would enable timely intervention and potentially prevent or delay the progression to chronic renal failure. It is also necessary to improve imaging techniques, such as specialized kidney magnetic resonance or ultrasound, to detect subtle changes [150,166].

### 4.6. Coenzyme Q10 Deficiency: Pathophysiology and Histopathological Changes Linked to Nephrotic Syndrome

Coenzyme Q10 (CoQ10), known as ubiquinone, is a lipid-soluble antioxidant that is one of the essential components of the mitochondrial electron transport chain, facilitating ATP production. Its key role is in the production of cellular energy, mainly through oxidative phosphorylation. In addition to the role it plays in energy metabolism, CoQ10 has pronounced antioxidant properties that protect cells from oxidative stress by removing free radicals. Therefore, CoQ10 deficiency, which results from genetic mutations, can lead to impaired mitochondrial function, increased oxidative damage, and cellular dysfunction. These disorders are particularly pronounced in organs that require energy for their work, such as the kidneys. One of the clinical manifestations in some patients with primary CoQ10 deficiency is SRNS [167,168,169,170,171].

Research has shown that several mechanisms can lead to CoQ10 deficiency, namely genetic mutations affecting CoQ10 biosynthesis, mitochondrial dysfunction, increased CoQ10 utilization due to oxidative stress, and nutritional insufficiency. CoQ10 deficiency can be (1) primary—a rare genetic disorder, caused by mutations in genes (COQ2, COQ4, COQ6, COQ7, COQ8A, COQ8B, and COQ9) involved in the synthesis of CoQ10 that disrupt the CoQ10 biosynthetic pathway; (2) secondary—occurs due to other underlying conditions, including mitochondrial disorders, use of statins (which inhibit the mevalonate pathway, precursor of CoQ10 synthesis), aging, as well as chronic diseases [167,172,173,174].

#### 4.6.1. Pathophysiology

In a healthy kidney, CoQ10 is involved in ATP synthesis, which is extremely important for numerous cellular functions, such as glomerular filtration, regulation of electrolyte balance, and tubular transport processes. Recent literature data suggest that the mechanisms responsible for the development of NS in the case of CoQ10 deficiency are multiple. Thus, as one of the mechanisms, damage to the function of mitochondria in podocytes, which have high energy needs and rely heavily on the production of mitochondrial ATP, is stated. CoQ10 deficiency can impair mitochondrial respiration in podocytes, which can further lead to reduced ATP synthesis and increased ROS production, which further predisposes podocyte damage. The described events disrupting the structure and function of podocytes eventually lead to proteinuria. As a possible mechanism for the formation of NS due to CoQ10 deficiency, studies report its influence on changes in the actin cytoskeleton in podocytes, which is necessary for maintaining their shape and function. Disruption of the actin cytoskeleton can lead to the loss of foot-like extensions of podocytes, which will result in proteinuria. In addition, it has been shown that CoQ10 deficiency can impair the synthesis and function of other GFR components, such as nephrin and podocin, thereby further contributing to proteinuria [167,171,172,175,176].

#### 4.6.2. Histopathological Changes

Analysis of kidney tissue biopsies from patients with NS associated with CoQ10 deficiency revealed changes in glomerular structure, podocytes, and tubulointerstitium. One of the significant changes seen by electron microscopy is the loss of foot-like extensions of podocytes. This analysis also reveals changes in the mitochondria of podocytes and tubular epithelial cells in the form of enlarged, pleomorphic mitochondria in which the presence of disorganized crystals is also observed. Interstitium fibrosis and tubule atrophy can be observed by light microscopy. Loss of tubular epithelial cells can occur due to apoptosis resulting from increased oxidative stress associated with low CoQ10 levels. Subsequently, an inflammatory reaction occurs that can further aggravate these changes [167,171,172].

#### 4.6.3. Clinical Implications and Therapeutic Interventions

The clinical presentation can vary—some patients may develop SRNS very early, while others may develop it later in life. In addition to the manifestations characteristic of NS, patients with CoQ10 deficiency may have other signs and symptoms, including muscle weakness, cardiac dysfunction, and neurological manifestations. The specific genetic mutation and degree of CoQ10 deficiency influence the severity of symptoms [173,177].

The diagnosis of CoQ10 deficiency requires a comprehensive approach. Initial screening should include determination of plasma and serum CoQ10 levels. However, as these measurements may not fully reflect the levels of CoQ10 in the tissue, especially in the kidneys, a definitive diagnosis of this disease requires a kidney biopsy to ensure a more accurate measurement of CoQ10 levels in the kidney tissue. In cases where CoQ10 deficiency is suspected, genetic testing is necessary to identify mutations in genes involved in CoQ10 biosynthesis and to confirm primary CoQ10 deficiency [178].

*Treatment* of NS associated with CoQ10 deficiency is based on CoQ10 supplementation and resolution of the NS. Most notable is CoQ10 supplementation with ubiquinone or ubiquinol, a reduced form of CoQ10 that studies have shown to be more effective. Restoration of CoQ10 levels with available CoQ10 formulations can lead to improvement of kidney function, reduction in proteinuria and mitigation of certain histopathological changes through improvement of mitochondrial function and reduction in oxidative stress. In addition to CoQ10 supplementation, it is necessary, as we have already mentioned, to include other therapeutic options that are aimed at the symptoms of NS. This includes diuretics, ACE inhibitors, ARB, and statins. In addition, vitamin E and vitamin C can be included in the treatment, which can protect kidney cells from damage by reducing oxidative stress [168,169,170,171,179,180].

#### 4.6.4. Future Directions and Research

Future research should focus on elucidating the precise mechanisms by which CoQ10 deficiency contributes to NS. A good understanding of the pathways of mitochondrial dysfunction, oxidative stress, and inflammatory responses, as well as the molecular pathways involved, could greatly facilitate the development of targeted therapies. Although the evidence presented is promising, further clinical studies are needed to evaluate and confirm the therapeutic efficacy of CoQ10 supplementation on NS. Furthermore, research should explore the potential of gene therapy to correct mutations in genes involved in CoQ10 biosynthesis. Gene therapy holds promise for restoring CoQ10 production and preventing the development of complications associated with CoQ10 deficiency. In order to make progress in therapy, it is necessary to conduct additional longitudinal studies that would follow patients over time to clarify the natural course of CoQ10 levels in relation to disease progression. In order to understand individual susceptibility to CoQ10 deficiency and NS, which would further help in the development of targeted therapy, we believe it is necessary to evaluate the role of gene polymorphisms that affect CoQ10 biosynthesis [178,181].

### 4.7. Monoclonal Gammopathy with Renal Significance: Pathophysiology and Histopathological Changes Linked to Nephrotic Syndrome

Monoclonal gammopathy (MG) is a group of disorders characterized by clonal proliferation of plasma cells or B lymphocytes, leading to excessive production of monoclonal immunoglobulins (M-proteins). Although the presence of M-protein is often asymptomatic, renal complications may occur in some patients. In such cases, we are talking about monoclonal gammopathy with renal significance (MGRS), which represents an exceptional challenge both for diagnosis and for treatment, considering that the pathophysiology and histopathological presentation can be very different [182,183].

#### 4.7.1. Pathophysiology

Different pathophysiological mechanisms can lead to renal manifestations in MGRS. The specific mechanism by which they will arise depends on the type of M-protein produced, its physicochemical properties, and interaction with kidney components. Three major categories of M-proteins can cause kidney damage, namely light chains (LCs), heavy chains (HCs), and intact immunoglobulins. Excessive production of LC (kappa or lambda) leads to their excessive filtration through the glomeruli. Under normal circumstances, free light chains (FLCs) are almost completely reabsorbed and catabolized in the proximal tubular cells. However, under overload conditions, this reabsorption capacity is exceeded, resulting in deposition of LC in the tubules, interstitium, or glomeruli. LCs can directly damage tubular cells, causing inflammation, dysfunction, and eventual fibrosis. LC deposition in distal tubules, especially in the presence of Tamm-Horsfall protein (uromodulin), can lead to the formation of cylinders and tubule obstruction, exacerbating interstitial inflammation and fibrosis. Kappa or lambda LC can interact with mesangial cells, inducing proliferation, matrix production, and release of inflammatory mediators. This interaction can lead to glomerular disease, such as amyloidosis or light-chain deposition disease (LCDD). Renal manifestations associated with HC are less frequent than those associated with LC. HC deposits can occur in the glomeruli, tubules, or interstitium, resulting in different patterns of kidney damage. The pathophysiological mechanisms associated with HC-induced kidney damage are not fully elucidated, but direct toxicity, complement activation, and the inflammatory response are believed to be responsible for its occurrence. Deposition of monoclonal intact immunoglobulins can lead to various types of glomerulonephritis, MN, membranoproliferative glomerulonephritis (MPGN), and fibrillary glomerulonephritis [182,183,184,185,186,187].

#### 4.7.2. Histopathological Changes

Some of the key renal manifestations of MGRS are as follows: (1) light-chain amyloidosis (AL amyloidosis), characterized by the deposition of LC-derived amyloid fibrils in the glomeruli, vascular structures, and interstitium. Immunoelectron microscopy reveals characteristic amyloid fibrils; (2) LCDD in which LCs are deposited linearly along the tubular basement membranes, GBM, and mesangium. Immunofluorescence reveals restriction for a specific LC isotype (kappa or lambda); (3) Monoclonal immunoglobulinemic glomerulonephritis (MIDD) representing various glomerular diseases characterized by the deposition of monoclonal Ig or their fragments. A variety of histologic patterns may occur, including MPGN, proliferative glomerulonephritis, and sclerosing glomerulonephritis; (4) light-chain-mediated proximal tubulopathy characterized by excessive reabsorption of LC in the proximal tubule that can lead to tubular dysfunction and damage, manifesting as Fanconi syndrome or acute tubular necrosis [187,188,189,190,191].

#### 4.7.3. Clinical Implications and Therapeutic Interventions

The *clinical* implications of MGRS are significant and complex. Renal involvement in patients with MGRS can profoundly affect overall prognosis and quality of life. The condition can occur in patients with multiple myeloma, chronic lymphocytic leukemia, or other malignancies associated with monoclonal proliferation. Recognition of the renal sequelae associated with these conditions is essential for timely treatment. Monitoring of renal function in individuals with known monoclonal gammopathy is recommended, as early intervention may be key in preventing irreversible damage. Furthermore, the systemic effects of nephrotic syndrome, such as susceptibility to infections and thromboembolic events, exacerbate the challenges faced by patients with MGRS [183,185,188,192].

*Evaluation of patients* with suspected MGRS requires a comprehensive diagnostic approach integrating clinical, laboratory, and pathologic findings. Initial laboratory tests usually include serum and urine protein electrophoresis to identify the monoclonal protein. Quantitative immunofixation can additionally provide insight into the specific type of monoclonal protein present (determination of serum FLC kappa and lambda, immunofixation electrophoresis, and serum protein electrophoresis). Renal function tests, which include serum creatinine and urinalysis for proteinuria, are fundamental in the assessment of kidney damage. In many cases, a kidney biopsy is required to confirm the diagnosis and understand the underlying histopathological changes. In doing so, it is extremely important to perform light microscopy in order to evaluate glomerular, tubular and interstitial changes; immunofluorescence analysis to detect and characterize deposits of Ig, LC and complement; and electron microscopy, which is necessary to identify ultrastructural features such as amyloid fibrils or crystalline inclusions [188,192].

Treatment of MGRS is primarily directed at treating the underlying clonal disorder, with measures to reduce M-protein production and alleviate kidney damage. Specific treatment strategies depend on the type of MG, the degree of kidney dysfunction, and the general health of the patient. Chemotherapy regimens are used for clonal plasma cells or B lymphocytes and to reduce M-protein production. In certain groups of patients with an underlying plasma cell malignancy, autologous stem cell transplantation can be considered. In patients with MGRS, it is necessary to include therapy aimed at controlling blood pressure, inhibition of ACE or ARB, as well as diuretics to control fluid overload [187,190,191].

#### 4.7.4. Future Directions and Research

Despite advances in the understanding and treatment of MGRS, several gaps in knowledge remain. Future research should aim to elucidate the precise mechanisms by which monoclonal proteins cause kidney damage, particularly with regard to specific nephron vulnerabilities. Understanding the genetic or environmental factors that may predispose certain populations to MGRS could also be beneficial. Large studies evaluating the long-term outcomes of patients with MGRS will provide key insights into disease progression and treatment efficacy. Improved diagnostic tools are needed, such as more sensitive and specific tests for the detection of monoclonal proteins. It is important to note that it is necessary to identify reliable biomarkers for the early diagnosis of renal involvement in patients with monoclonal gammopathy and to monitor the response to treatment [186,187,189,190].

The genetic basis, primary pathophysiological mechanisms, secondary effects, and key histopathological changes are detailed in Table 1.

Additionally, the clinical manifestations and therapeutic approaches related to these syndromes are summarized in Table 2.

Biomarkers for the diagnosis of rare genetic and metabolic disorders that affect the kidneys and therefore manifest as nephrotic syndrome, as well as the importance of accurate diagnostic biomarkers, are shown in Table 3 and Table 4.

## 5. Challenges in the Research of Rare Genetic Disorders

Conditions such as Schimke syndrome, LCAT deficiency, FED, familial LCAT deficiency caused by mutations such as p.Leu364Pro, nephrotic syndrome coexisting with type 1 diabetes, CDG, NPS, CoQ10 deficiency, and MGRS are individually rare, making patient cohorts small and geographically dispersed. The research of rare genetic and metabolic disorders associated with NS represents a great challenge, and further progress in their study is greatly hindered precisely by their rarity. Given this fact, the lack of relevant data for a meaningful statistical analysis is completely understandable.

The power to detect significant associations between genetic mutations and disease outcomes is limited by small sample sizes. Difficulties in the diagnosis and treatment of these conditions certainly carry highly variable clinical manifestations. Another factor that makes it difficult to diagnose these conditions is the overlap of symptoms with more common kidney diseases. All this certainly leads to a delayed diagnosis, both in terms of the manner and effectiveness of treatment. Understanding the precise mechanisms by which these genetic defects lead to NS requires advanced research techniques, including genetic sequencing, cell culture studies, and research in animal models. This can be extremely difficult because the human disease phenotype must be fully replicated.

That is why we believe that the priority of international cooperation in research should be the pooling of data and resources. In this way, the limitations of a small number of patients could be overcome. New, modern genomic technologies, such as exome sequencing, are necessary to identify new genetic changes and to understand the mechanisms of these associated diseases. It is necessary to work intensively on the development of targeted therapies, such as gene therapy or personalized medical approaches. For families affected by these conditions, genetic counseling is necessary to provide relevant information about inheritance patterns, recurrence risks, and potential reproductive options. In accordance with all of the above, it is clear that a multidisciplinary approach is necessary to solve the complex needs of patients.

Therefore, as we face serious difficulties in conducting clinical studies and generating sufficient statistical data, and since the heterogeneity of these diseases complicates research efforts because it is difficult to identify clear correlations between genotype and phenotype, we believe that a multidisciplinary approach, genetic testing and recognition of histopathology are essential. Therefore, international cooperation is very important, which includes the following: (1) development of international registries to collect data on rare diseases affecting the kidneys and manifesting as NS; (2) establishment of biobanks for tissue samples and genetic samples to facilitate research; (3) coordination of research efforts in multiple centers to collect adequate sample sizes; (4) opening platforms for the exchange of research findings, clinical experiences and treatment outcomes; (5) further improvement of genetic testing technologies with increased sensitivity and specificity of diagnostics, which would certainly lead to the development of new therapeutic agents.

## 6. Conclusions

In addition to more common causes such as MCD and FSGS, there are specific, rare diseases and genetic disorders that can cause the development of NS. Examples of such causes include genetic, inherited diseases such as Schimke syndrome, fish-eye disease, and Nail–Patella Syndrome. Moreover, NS can also occur in people with T1D, which requires a special diagnostic approach. Less common are genetic mutations such as the p.Leu364Pro mutation that has been reported in familial LCAT deficiency. In suspected cases, it is crucial to consider genetic analysis and a thorough clinical examination to ensure a timely and accurate diagnosis. Detailed tests, including biopsy, genetics tests and a multidisciplinary approach, are required. Recognizing these rare causes is extremely important both for an accurate diagnosis and for successful treatment, that is, more effective treatment that includes the application of targeted therapy and a better prognosis.

## Figures and Tables

**Table 1:** Comparative analysis of syndromes associated with nephrotic syndrome.

Disorder	Genetic Basis	Primary Pathophysiological Mechanism	Secondary Effects	Key Histopathological Changes
Schimke Syndrome	SMARCAL1 gene mutation	Disorder of chromatin remodeling and gene expression	Genomic instability, DNA fragmentation	FSGS; podocyte infolding glomerulopathy
Lecithin-Cholesterol Acyltransferase (LCAT) Deficiency—fish-eye disease	Partial deficiency of LCAT	Disorder of lipoprotein metabolism	Accumulation of non-esterified cholesterol	Foam cells, GBM thickening, mesangial proliferation, lipid vacuoles in endothelial cells
Lecithin-Cholesterol Acyltransferase (LCAT) Deficiency—familial form	p.Leu364Pro mutation	Disorder of lipoprotein metabolism	Complete lack of LCAT enzyme	Thickening of capillary walls, foam cells, lacunar spaces in the GBM and electron-dense deposits in the mesangium
Nephrotic Syndrome Co-existing with Type 1 Diabetes	HLA antigens A24, DR4, DR53	Immunological dysregulation	Oxidative stress, inflammation	MCD in the early stages, diffuse glomerulosclerosis in the later stages
Congenital Disorders of Glycosylation	Mutations of PMM2, ALG1, ALG6, ALG3	Disorder of protein N-glycosylation	Disorder of protein folding and function	Diffuse mesangial sclerosis
Nail–Patella Syndrome	Mutations of the LMX1B gene	Disorder of podocyte development	Abnormal cleft diaphragms	GBM “eaten by moths”
Coenzyme Q10 deficiency	involved in CoQ10 biosynthesis (COQ2, COQ4, COQ6, COQ7, COQ8A, COQ8B, COQ9)	Impaired mitochondrial function and energy production	Increased oxidative stress, disruption of podocyte cytoskeleton	Podocyte foot process effacement, abnormal mitochondria, interstitial fibrosis
Monoclonal gammopathy with renal significance	Clonal proliferation of plasma cells or B lymphocytes (not primarily genetic)	Excessive production and deposition of monoclonal immunoglobulins	Direct toxicity, complement activation, inflammatory response	Amyloid fibrils, light-chain deposits, heavy-chain deposits, glomerular lesions, tubular damage

GBM—glomerular basement membrane; FSGS—focal segmental glomerulosclerosis; MCD—minimal change disease; Q10—Coenzyme Q10; SMARCAL1—SWI/SNF-related, matrix-associated, actin-dependent regulator of chromatin, subfamily A-like 1; LCAT—Lecithin-Cholesterol Acyltransferase; HLA—Human Leukocyte Antigen; PMM2—Phosphomannomutase 2; ALG1—asparagine-linked glycosylation 1; ALG6—alpha-1,3-glucosyltransferase; ALG3—alpha-1,3-mannosyltransferase; LMX1B—LIM homeobox transcription factor 1-beta.

**Table 2:** Comparative analysis of syndromes associated with nephrotic syndrome—clinical manifestations and therapeutic approach.

Syndrome	Renal Manifestations	Extrarenal Manifestations	Age	Therapeutic Approach
Schimke syndrome	Steroid-resistant nephrotic syndrome	Growth retardation, spondyloepiphyseal dysplasia, defect in T-cell immunity	Childhood	Corticosteroid therapy (often resistant). Immunosuppressive drugs. Symptomatic therapy. Kidney transplantation in terminal renal failure.
LCAT deficiency—fish-eye disease	Mild to severe proteinuria	Corneal clouding, dyslipidemia	Adolescence/adulthood	Control of lipid status. Statins and other hypolipemic agents. Plasmapheresis. Enzyme replacement therapy (experimental). Kidney transplantation in terminal renal failure.
LCAT deficiency—familial form	Severe proteinuria, progressive renal failure	Corneal clouding, anemia, dyslipidemia	Childhood/adolescence	Control of lipid status. Statins and other hypolipemic agents. Plasmapheresis. Enzyme replacement therapy (experimental). Kidney transplantation in terminal renal failure.
Nephrotic Syndrome Co-existing with Type 1 Diabetes	Proteinuria, edema, hypoalbuminemia	Hyperglycemia, polyuria, polydipsia, weight loss	Variable	Glycemic control (insulin). Corticosteroid therapy for nephrotic syndrome. ACE inhibitors or ARBs for proteinuria. Blood pressure control.
Congenital Disorders of Glycosylation	Nephrotic syndrome in infancy or early childhood	Neurological disorders, skeletal system abnormalities, liver dysfunction	Infancy/early childhood	Specific therapy depending on the type of CDG (eg mannose supplementation for PMM2-CDG). Symptomatic therapy. Dietary interventions.
Nail–Patella Syndrome	Proteinuria with or without hematuria, progressive renal failure	Dysplasia of the nails, hypoplasia of the patella, deformity of the elbows, involvement of the eyes	Variable	ACE inhibitors or ARBs for proteinuria. Blood pressure control. Symptomatic therapy. Kidney transplantation in terminal renal failure.
CoQ10 Deficiency	Steroid-resistant nephrotic syndrome, proteinuria	Muscle weakness, cardiac dysfunction, neurological manifestations	Can present early in life or later, depending on mutation	CoQ10 supplementation, symptomatic treatment of NS
Monoclonal Gammopathy with Renal Significance	Various patterns: amyloidosis, LCDD, glomerulonephritis, tubulopathy	Varies based on underlying condition (multiple myeloma, CLL, etc.)	Typically affects older adults	Treatment of underlying clonal disorder, supportive therapy

LCAT—Lecithin-Cholesterol Acyltransferase; LCDD—Light Chain Deposition Disease, CLL—Chronic Lymphocytic Leukemia, ACE—Angiotensin-Converting Enzyme, ARBs—Angiotensin II Receptor Blockers, CDG—Congenital disorder of glycosylation, PMM2-CDG—Phosphomannomutase 2-Congenital Disorder of Glycosylation, CoQ10—Coenzyme Q10, NS—nephrotic syndrome.

**Table 3:** Overview of key diagnostic biomarkers for rare syndromes that manifest with nephrotic syndrome.

Syndrome	Specific Biomarkers	Nonspecific Biomarkers
Schimke syndrome	SMARCAL1 expression in kidney biopsy	Proteinuria, hypoalbuminemia, hyperlipidemia
LCAT deficiency	Activity LCAT plasma enzymes, abnormal lipid profile (↑ Unregistered cholesterol, ↓ HDL)	Proteinuria, hypoalbuminemia
Nephrotic Syndrome Co-existing with Type 1 Diabetes	Anti-GAD antibodies, HLA typing (A24, DR4, DR53)	Hyperglycemia, glycosuria, proteinuria
Congenital glycosylation disorders	Abnormal transferrin pattern (isoelectric focus), specific enzyme activity	Proteinuria, hypoalbuminemia
Nail–Patella Syndrome	Expression of LMX1B in the kidney biopsy	Proteinuria, Hematuria
CoQ10 deficiency	Levels of coenzyme Q10 in serum	Mitochondrial function tests
MGRS	Protein electrophoresis, light chains	Serum creatinine

CoQ10—Coenzyme Q10; MGRS—monoclonal gammopathy with renal significance. LCAT—Lecithin-Cholesterol Acyltransferase; SMARCAL1—SWI/SNF-related, matrix-associated, actin-dependent regulator of chromatin, subfamily A-like 1; GAD—Glutamic Acid Decarboxylase, HLA—Human Leukocyte Antigen.

**Table 4:** Importance of accurate diagnostic biomarkers.

Clinical Benefits	Research Implications
Facilitates timely diagnosis	Enhances understanding of disease mechanisms
Informs treatment decisions	Identifies potential therapeutic targets
Improves patient outcomes	Supports development of novel treatments
Enables personalized medicine approaches	Facilitates clinical trial design and participant selection

## Data Availability

No new data were created or analyzed in this study. Data sharing is not applicable to this article.

## Abbreviations

The following abbreviations are used in this manuscript:

NS	Nephrotic syndrome
MCD	Minimal change disease
FSGS	Focal segental glomerulosclerosis
DN	Diabetic nephropathy
CNS	Cogenital nephrotic syndrome
GBM	Glomerular basement membrane
MN	Membranous nephropathy
SLE	Systemic lupus erythematosus
GFB	Glomerular filtration barier
SSNS	Steroid-sensitive nephrotic syndrome
SRNS	Steroid-resistant nephrotic syndrome
SIOD	Schimke immuno-osseous dysplasia
LCAT	Lecithin-cholesterol acyltransferase
FED	Fish-eye disease
HDL	High-density lipoprotein
LDL	Low-density lipoprotein
HLA	Human leukocyte antigen
T1D	Type 1 diabetes
CDG	Congenital disorders of glycosylation
AS	Alport syndrome
HBI	Chronic renal failure
DM	Diabetes mellitus
NSAIL	Nonsteroidal anti-inflammatory drugs
PIG	Podocyte infolding glomerulopathy
NPS	Nail–Patella Syndrome
ESRD	End-Stage Renal Disease
ACE	Angiotensin-converting enzyme inhibitors
ARB	Angiotensin receptor blockers
GLP-1	Glucagon-like peptide-1
SGLT2	Sodium-glucose co-transporter 2
CoQ10	Coenzyme Q10
MGRS	Monoclonal gammopathy with renal signficance
MG	Monoclonal gammopathy
LC	Light chains
HC	Haevy chains
FLC	Free light chain
LDCC	Light chain deposition disease
MPGN	Membranoproliferative glomerulonephritis
MIDD	Monoclonal immunoglobulinemic glomerulonephritis
